# Influence of COVID-19 on the 10-year carbon footprint of the Nagoya University Hospital and medical research centre

**DOI:** 10.1186/s12992-022-00883-9

**Published:** 2022-11-07

**Authors:** Hikaru Morooka, Takanori Yamamoto, Akihito Tanaka, Kazuhiro Furuhashi, Yasuhiro Miyagawa, Shoichi Maruyama

**Affiliations:** 1 Department of Emergency and Critical Care Medicine, Nagoya University Graduate School of Medicine, Tsurumai-cho, 65, Showa Ward, 466-8560 Nagoya, Aichi Japan; 2 Department of Public Health and Nursing, Norwegian University of Science and Technology, N-7491 Trondheim, Norway; 3 Department of Nephrology, Nagoya University Hospital, Tsurumai-cho, 65, Showa Ward, 466-8560 Nagoya, Aichi Japan; 4 Department of Hospital Pharmacy, Nagoya University School of Medicine, Tsurumai-cho, 65, Showa Ward, 466-8560 Nagoya, Aichi Japan; 5 Division of Nephrology, Nagoya University Graduate School of Medicine, Tsurumai-cho, 65, Showa Ward, 466-8560 Nagoya, Aichi Japan

**Keywords:** Carbon footprint, COVID-19, Greenhouse Gas Protocol, Hospitals, Carbon emissions

## Abstract

**Background:**

Amidst the climate crisis, a key goal of the medical sector is to reduce its large carbon footprint. Although the Coronavirus disease 2019 (COVID-19) pandemic greatly impacted the medical sector, its influence on carbon footprints remains unknown. Therefore, the aim of this study was to evaluate changes in the carbon footprint of a university hospital with a medical research centre over the past 10 years.

**Methods:**

Data on electricity, gas, and water usage, pharmaceutical and medical supply costs, and waste amounts were recorded for Nagoya University Hospital from April 2010 to March 2021. The relevant emission factors were obtained from the Japanese government and the overall monthly carbon footprint was reported according to the Greenhouse Gas Protocol. The effect of the COVID-19 pandemic on the carbon footprint was then compared for three types of emission sources. Moreover, a regression model was used to plot quadratic functions as approximate functions using monthly carbon emissions and monthly average external temperatures. Finally, the monthly carbon footprint was calculated per hospital admission.

**Results:**

The overall carbon footprint of the hospital was 73,546 tCO_2_e in 2020, revealing an increase of 26.60% over the last 10 years. Carbon emissions from electricity consumption represented 26% of total emissions. The individual carbon footprints of pharmaceuticals, medical supplies, waste, and water usage also increased from 2010 to 2020. The overall monthly carbon footprint was positively correlated with the average monthly temperature (R^2^ = 0.7566, p < 0.001). Compared with 2019, the overall carbon footprint decreased by 2.19% in 2020. Moreover, the monthly carbon footprint per hospital admission increased significantly between 2018 (0.24 tCO_2_e/admission) and 2020 (0.26 tCO_2_e/admission) (p = 0.002).

**Conclusion:**

The overall carbon footprint of the hospital generally increased over the last decade. During the COVID-19 epidemic in 2020, the carbon footprint decreased slightly, likely because of the reduced number of patients. However, the carbon footprint per admission increased, which was attributed to more complicated patient backgrounds because of the ageing population. Therefore, evaluation of carbon emissions in the medical sector is urgently required in order to act on the climate crisis as soon as possible.

**Supplementary Information:**

The online version contains supplementary material available at 10.1186/s12992-022-00883-9.

## Background

Climate change has become an increasingly critical problem affecting all humans on Earth. Despite implementation of Agenda 21 and Agenda 30 in 1992 and 2015, respectively, the climate crisis remains one of the greatest threats to human life [1, 2]. The medical industry currently accounts for a large proportion of the carbon footprint of developed countries, for example, 10% of the carbon footprint in the USA [3]. However, the climate crisis is likely to increase the number of people requiring healthcare as a result of heat stroke, forest fires, and storms [4]. Therefore, the goal of many countries is to reduce carbon emissions from the medical sector, for example, the UK National Health Service is committed to becoming carbon neutral by 2045 [5].

As the sixth largest emitter of greenhouse gases in 2019, Japan represents a major contribution to the global carbon footprint [6]. Moreover, as a developed country with a history of numerous industrial pollution events, such as Yokkaichi asthma, Minamata disease, and Itai-itai disease, Japan has proposed several initiatives on climate change action [7–9]. For example, Japan adopted the Kyoto Protocol in 1997, which extended Agenda 21 [10], and has committed to reducing its greenhouse gas emissions by 26% from 2013 levels by 2030, with the aim of becoming carbon neutral by 2050 [11, 12]. The carbon footprint of the Japanese healthcare system accounts for 5% of the total domestic carbon footprint [13]; thus, it is imperative to reduce carbon emissions from the medical sector to meet the goal of carbon neutrality by 2050 [12]. To enable this, it is important to monitor the current state and trajectory of carbon emissions in this sector.

Coronavirus disease 2019 (COVID-19) has placed a tremendous burden on public health [14]. Furthermore, the environmental effects of COVID-19 cannot be overlooked. Since the beginning of the COVID-19 pandemic, medical professionals have been using more personal protective equipment (PPE), performing increased ventilation of air inside buildings, and maintaining greater physical space between people to prevent infection [15]. Thus, COVID-19 has evidently changed medical practices. The practice of social distancing, i.e. limiting human activities to protect oneself from COVID-19, reportedly led to a reduction in carbon emissions in Seoul, South Korea [16, 17]. However, the environmental burden of the COVID-19 pandemic remains unclear, especially regarding carbon emissions from healthcare settings.

Therefore, the aim of this study is to determine the temporal variation in the carbon footprint of a university hospital with a medical research centre over the past 10 years. Specifically, we evaluate how the total carbon footprint of the Nagoya University Hospital (NUH) campus was impacted by the COVID-19 pandemic. The following hypotheses were tested in this study. Hypothesis 1 proposes that the overall carbon footprint of NUH has increased over the last 10 years, considering that Japan has an ageing society, which contributes to increased medical budgets and greater demand for medical resources [18]. Hypothesis 2 proposes that the monthly average external temperature is correlated to the monthly carbon footprint. Hypothesis 3 proposes that the carbon footprint decreased during the COVID-19 pandemic. This study makes the following novel contributions to the research field. First, this is the first study to analyse the total carbon footprint over a period of 10 years in a medical setting. Second, relevant local emission factors are used to more accurately calculate the carbon footprint. Third, we estimate changes in the carbon footprint of a large university hospital with a research centre during COVID-19. In the following sections, we describe the data collection and methods, including sensitivity and statistical analyses, present the results, discuss the implications of the results, and then present the conclusions of this study.

## Methods

### Theoretical framework

**Fig. 1:**
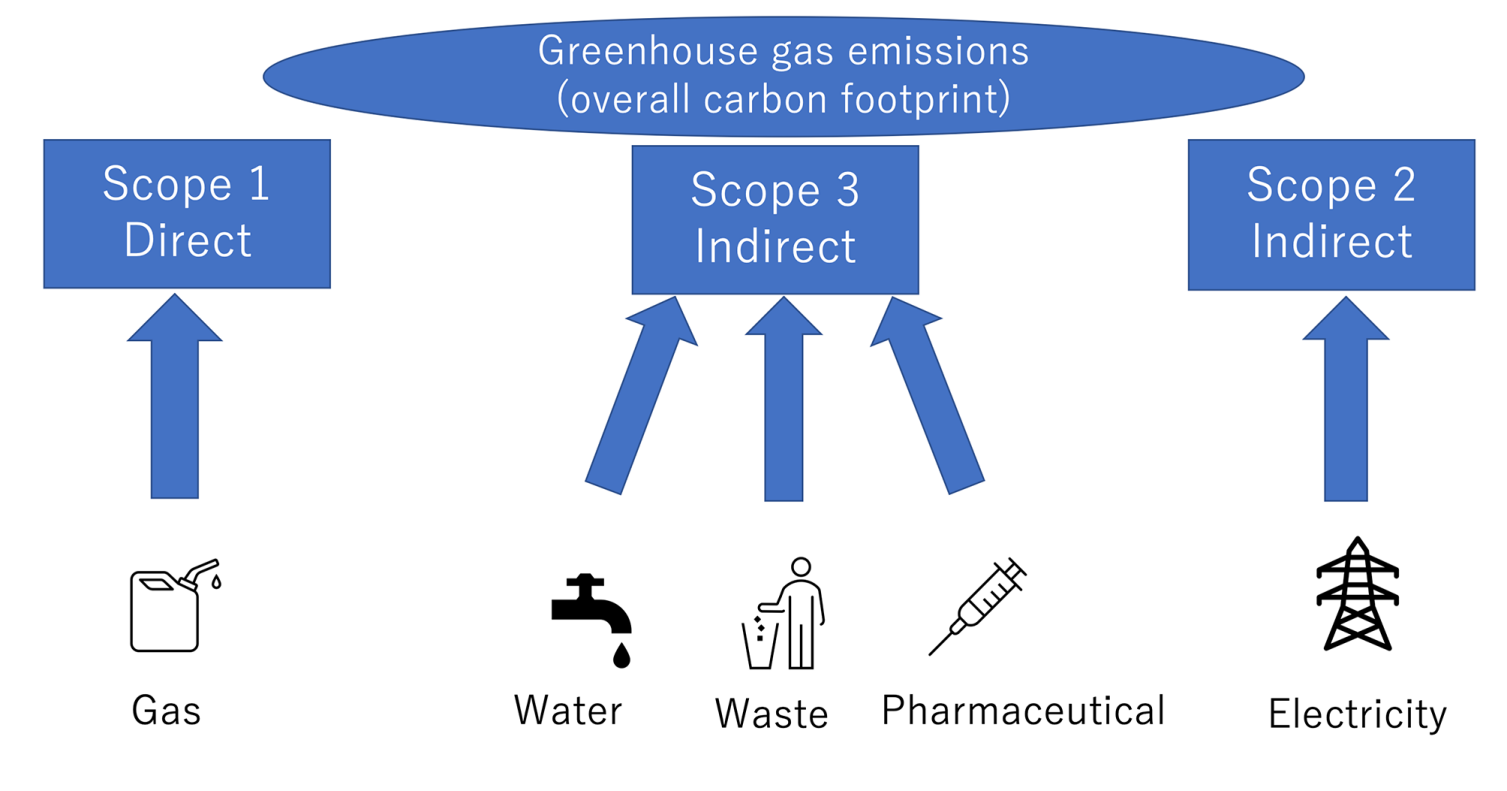
Diagram of the study protocol

In this study, the Greenhouse Gas (GHG) Protocol was used to assess the carbon footprint [19–21]. In the GHG protocol, carbon emission sources are divided into three scopes: scope 1 for direct carbon emissions from the campus; scope 2 for indirect emissions from the generation of purchased energy; and scope 3 for other indirect emissions occurring in the value chain of the campus (Fig. 1). This study followed the same methodology as previous studies from both medical and non-medical fields [22–25]; thus, scope 1 was defined as the carbon footprint from gas usage, scope 2 was the carbon footprint from electricity usage, and scope 3 was the carbon footprint from pharmaceutical and medical supplies, waste, and water usage (Fig. 1). The overall carbon footprint at NUH combines scope 1, 2, and 3. These variables are typically easy to obtain for hospitals because they are important financial indicators. For this 10-year longitudinal study, data were collected from 2010 to 2020.

### Emission factor data

To calculate the carbon footprint, it is necessary to derive both the original consumption data and the emission factors. The GHG protocol provides country specific emission factors, which are crucial for calculating accurate carbon emissions, for China, Mexico, and India, but not Japan [26]. However, the Japanese Ministry of the Environment provides lists of emission factors for the GHG protocol [27–29]. In this study, we used the emission factors of the Japanese Ministry of the Environment for gas, waste, and the cost of pharmaceuticals and medical supplies, because these should not differ with location. Because the emission factors for the cost of pharmaceuticals and medical supplies are calculated by lifecycle assessment, which includes greenhouse gas emissions through the supply chain [30], we excluded anaesthetic gases from the calculation in this study.

Conversely, the emission factors for electricity and water depend on the characteristics of each region. For example, regions with more water resources have an electricity supply that is more dependent on hydrogen power than other regions, leading to lower emission factors because of less petrol use. Regarding electricity emission factors for Nagoya City, Japan, Chubu Electric Power Co., Inc. publishes its emission factors for different years on its website [31, 32]. Annual local emission factors for clean water and sewage from April 2010 to March 2021 were obtained from Nagoya City Waterworks & Sewerage Bureau [33] via a questionnaire. All emission factors are shown in Additional Table 1 .

### Data collection

The Japanese government requires each office to report its annual carbon footprint [34]. Therefore, a hospital such as NUH must report its annual carbon footprint to the government, which is based on electricity and gas usage only, thereby excluding crucial aspects of the carbon footprint. NUH has several monitors for electricity and gas inside the campus, which are checked by the electricity and gas companies, who charge NUH a fee every month. Because of this, NUH has stored its monthly electricity and gas usage data for the last 10 years. Moreover, NUH maintains detailed accounts of its fees for waste, water, and the cost of pharmaceuticals and medical supplies. Every month, NUH is charged for its waste after a company measures each type of waste by weight. Information on waste types, including solid waste, scrap metal, and medical waste (both infectious and non-infectious) are available from April 2014. Every 2 months, the water monitor at NUH is checked to determine the fee for water consumption. Therefore, bimonthly water consumption data were divided by two to obtain the data for one month. Moreover, NUH monitors the cost of pharmaceuticals and medical supplies on an annual basis; therefore, these data were corrected to obtain monthly data. The number of hospitalised patients and outpatients is monitored once per month from electronic medical records. Specifically, NUH monitors the number of occupied beds and the monthly average hospital stay. All of the above data were provided by the NUH administration office. As this study does not use patients’ private information, an ethical review was unnecessary. The study was approved by NUH. Monthly average external temperature data for Nagoya City were obtained from the Japan Meteorological Agency [35].

### Experimental setting

NUH, one of the largest hospitals in Japan, is located in Tsurumai, Nagoya City. The hospital is a hub of clinical and preliminary research in Japan, with over 1,000 beds, including 50, 40, and 10 psychiatric, intensive care unit, and neonatal intensive care beds, respectively. The NUH campus includes two residency buildings for nurses, nine research and educational buildings, and eight hospital buildings. In 2021, the campus had approximately 150 teaching staff. The hospital employs over 2,000 staff and receives over 500,000 outpatients annually. COVID-19 first affected Nagoya around March 2020, with the hospital and campus changing their policy to reflect the pandemic situation in April 2020. Therefore, we defined the period prior to April 2020 as pre-COVID-19.

### Sensitivity analysis

For the sensitivity analysis, both annual and monthly carbon footprint data were calculated per hospital admission and per occupied bed from April 2018 to March 2021. NUH started to use its new clinical building fully from April 2018; therefore, this study period was employed to ensure that we only compared relatively similar situations. The carbon footprint per admission was employed to represent the quality of the patient’s medical care, whereas the carbon footprint per occupied bed was employed to represent the systematic carbon footprint of NUH. However, because NUH includes education, clinical work, and research, the results of this analysis should be interpreted with care.

### Statistical analyses

The energy consumption and carbon footprint data were compared using the one-way ANOVA or χ^2^ test. A regression model was used to plot quadratic functions as approximate functions using monthly carbon emissions and average temperature data, and the coefficient of determination (R^2^) was calculated to determine the relationship between these two variables. Kruskal–Wallis tests were conducted to determine the statistically significant differences between two or more groups, each category was tested with the Dunn test, and Bonferroni correction was used to adjust the p-value. Statistical significance was set to p < 0.05. All statistical analyses were performed using R (version 4.1.2, R Foundation for Statistical Computing, Vienna, Austria; https://www.r-project.org).

## Results

### Hypothesis 1: the overall carbon footprint of NUH has increased over the last 10 years

**Fig. 2:**
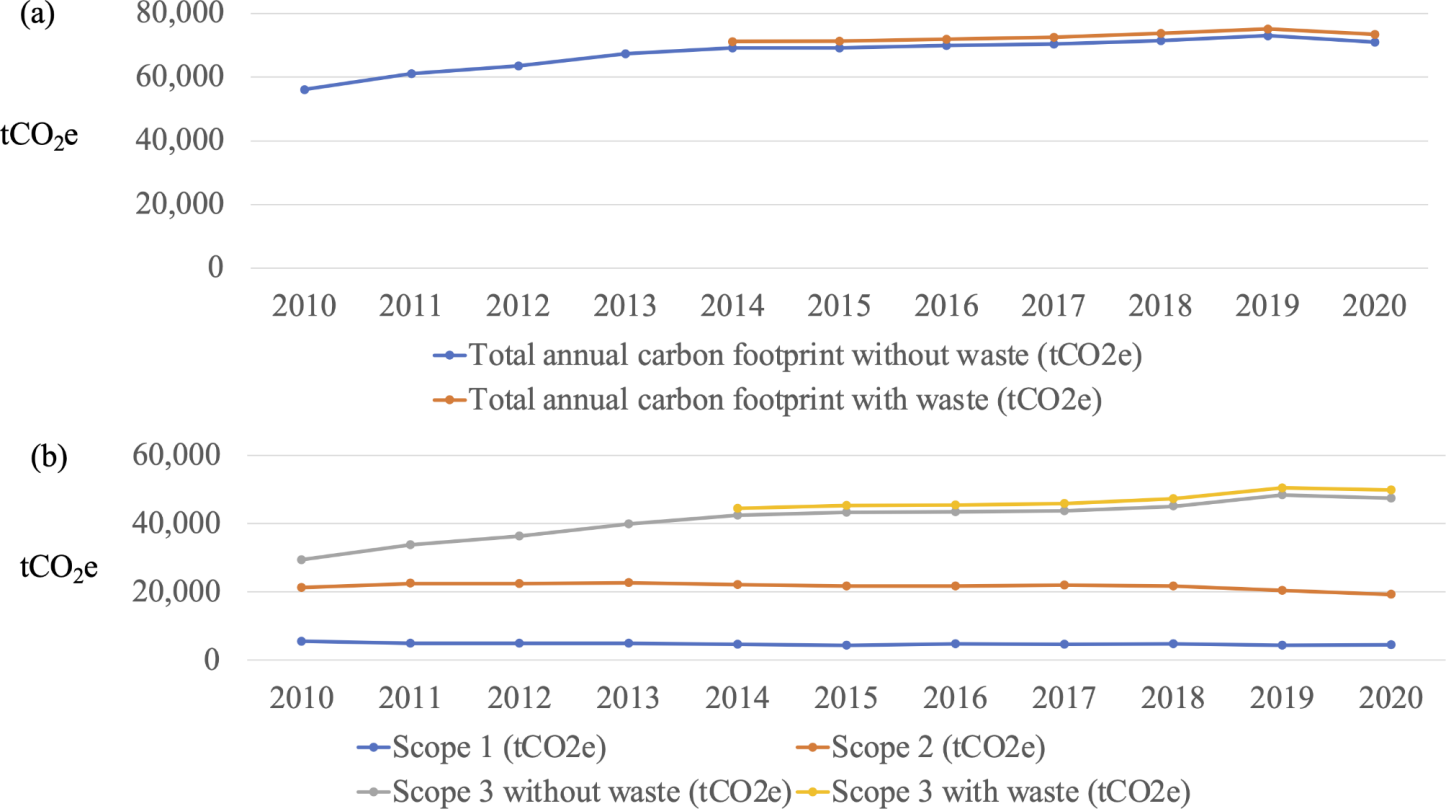
Total annual carbon footprint of Nagoya University Tsurumai Campus from 2010 to 2020 (a) with and without waste and (b) for each emission scope

Figure 2a represents the overall carbon emissions, with and without waste, at the NUH campus. The highest carbon emissions (75,200 tCO_2_e) were observed in 2019. The carbon footprint showed an increasing trend over the study period, except in 2020. Additional Table 2 presents the annual median carbon footprints from 2010 to 2020. Moreover, the carbon footprint decreased from 2019 to 2020, with a monthly median carbon footprint for the NUH campus of 6,209.19 tCO_2_e [interquartile range: 6,139.21, 6,294.47] in 2019 and 6,057.13 tCO_2_e [interquartile range: 5,975.74, 6,277.47] in 2020.

### Comparison of carbon emissions among the three scopes

Figure 2b presents the annual carbon emissions of each scope. Most carbon emissions fall under scope 3. The carbon emissions under scope 3 increased over the study period, reaching 50,000 tCO_2_e in 2019. The carbon emissions under the other two scopes were stable over the 10-year period, at approximately 5,000 tCO_2_e in scope 1 and less than 25,000 tCO_2_e in scope 2. However, the carbon emissions under scope 2 exhibited a slight decrease since 2017. Additional Table 2 shows the median monthly carbon footprint of each scope. In scope 1, minor changes were observed over the study period, but the carbon footprint remained relatively stable within a range from 345 tCO_2_e to 425 tCO_2_e (Additional Fig. 1 a). The carbon footprint of scope 2 (Additional Fig. 1 b) increased from 2010 to 2013 and in 2017, then gradually decreased after 2017. As waste data were not available until 2014, scope 3 was divided into the carbon footprint with waste and that without waste. In scope 3 (Additional Fig. 1 c), the monthly median carbon footprint both with and without waste increased over the study period, reaching up to 4,200 tCO_2_e in 2019 (with waste).

### Carbon emissions within each scope

No significant differences were observed in the monthly median electricity and gas usage from 2010 to 2020 (p = 0.178 and 0.570, respectively, Additional Table 2). However, annual electricity usage increased after 2016 (Additional Fig. 2 ), which was attributed to use of the new NUH clinical building from approximately 2017. Annual gas usage gradually decreased from 2010 to 2020 (Additional Fig. 3 ). The monthly median carbon emissions resulting from electricity use showed a significant difference (p = 0.024) over the study period, with a decreasing trend from 2017. However, no significant differences were observed in the carbon footprint of monthly median gas usage from 2010 to 2020 (p = 0.570, Additional Table 2). The usage and carbon footprint of clean water and sewage exhibited similar trends over the study period, except in 2020. The annual usage of clean water and sewage decreased from 2010 to 2013, was stable from 2014 to 2017, increased until 2019, then decreased again in 2020 (Additional Fig. 4 a), whereas the monthly median carbon footprint of clean water and sewage gradually decreased until 2017, increased in 2018, then decreased again in 2020 (Additional Fig. 4 b). The annual usage, monthly median usage, and monthly median carbon footprint of solid waste were stable from 2014 to 2017, increased in 2018, then decreased again after 2018 (Additional Fig. 5a; Additional Table 2); the same values for scrap metal exhibited a gradually decrease over the study period (Additional Fig. 5b and Additional Table 2). Medical waste showed different trends. First, the amount of non-infectious medical waste gradually decreased over the study period, whereas the amount of infectious medical waste gradually increased, except for an apparent spike in 2020 (p < 0.001; Additional Fig. 6c and Additional Table 2). Second, the amount of both solid waste and scrap metal decreased in 2020 (Additional Fig. 6a and b).

**Fig. 3:**
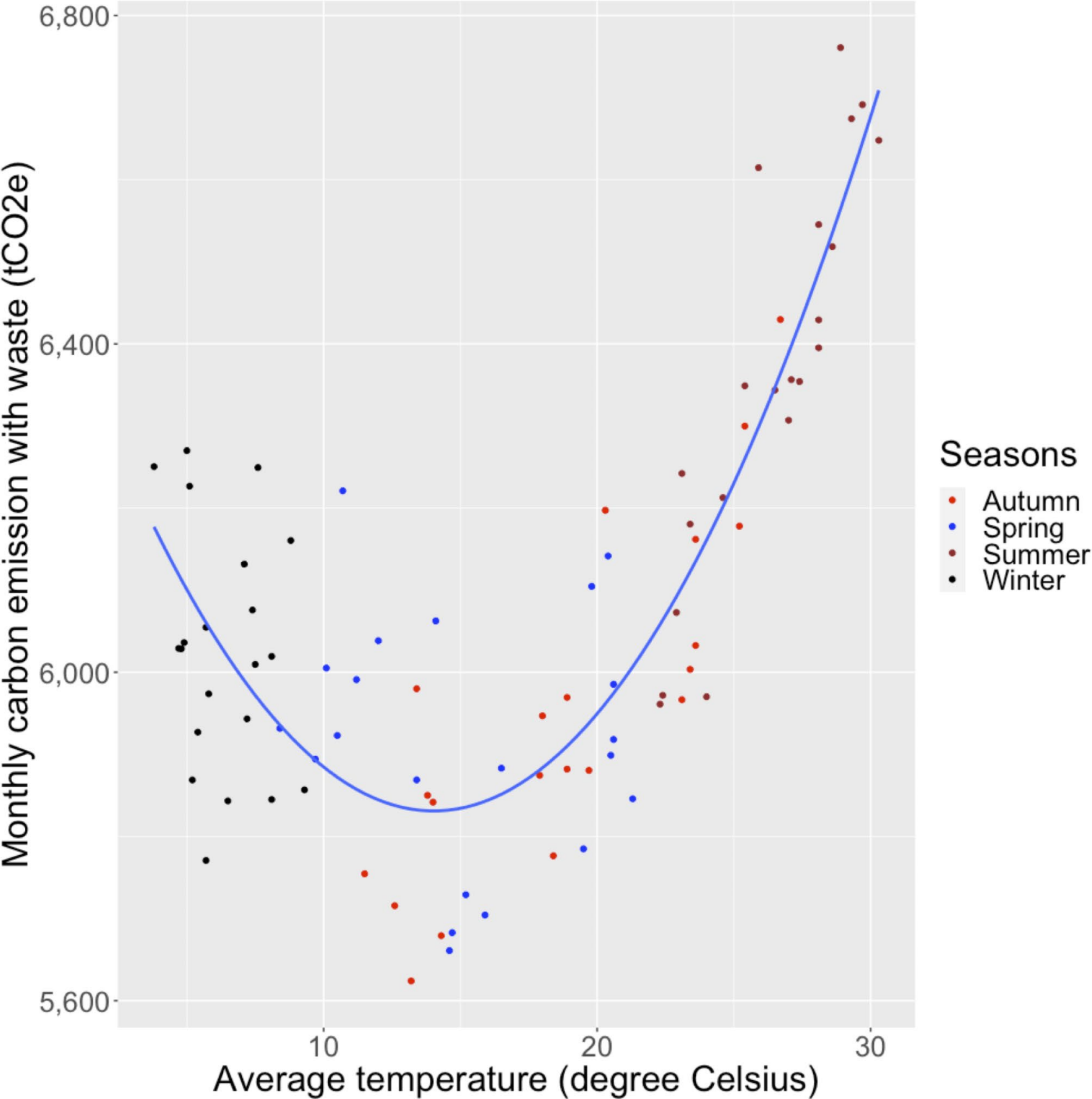
Correlation between monthly average external temperature and monthly carbon footprint from April 2014 to March 2021. Autumn: September, October, November. Spring: March, April, May. Summer: June, July, August. Winter: December, January, February

**Fig. 4:**
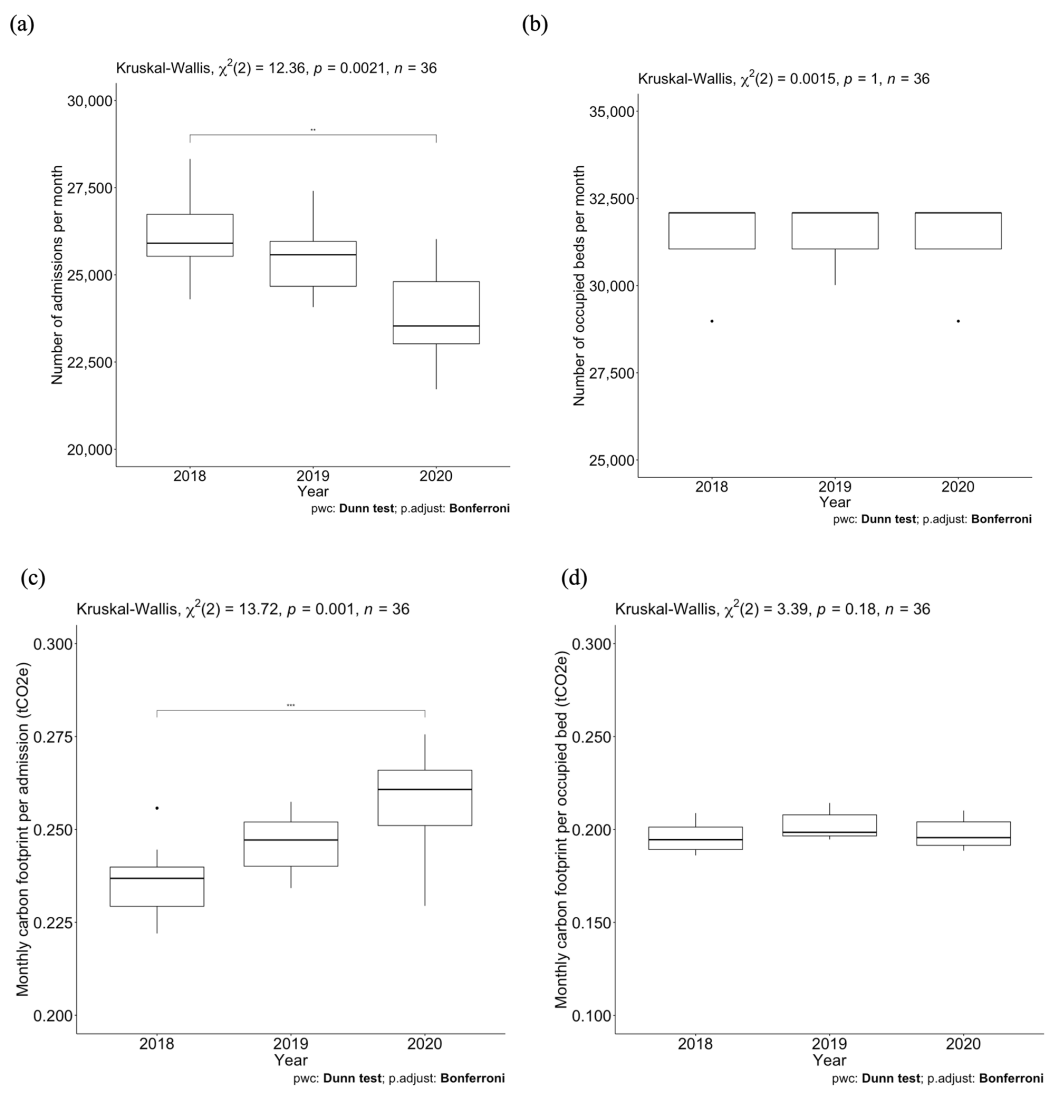
Kruskal–Wallis test plots from 2018 to 2020 showing (a) the number of hospital admissions per month, (b) the number of occupied beds per month, (c) the monthly carbon footprint per hospital admission, and (d) the monthly carbon footprint per occupied bed

### Hypothesis 2: monthly average external temperature is correlated to the monthly carbon footprint

Figure 3 shows the correlation between monthly average temperature and monthly carbon emissions. The R^2^ value was greater than 0.70 (R^2^ = 0.7452, p < 0.001), indicating a positive correlation between monthly average temperature and monthly carbon emissions. A similar trend was observed between monthly average temperature and electricity usage, emissions from electricity usage, and emissions from gas usage, with R^2^ values of 0.873, 0.8073, and 0.8186, respectively (Additional Figs. 7 and 8, and 9). However, the monthly average temperature exhibited no correlated with the emissions from clean water usage, sewage, or waste (Additional Figs. 10 and 11, and 12).

### Hypothesis 3: the carbon footprint decreased during the COVID-19 pandemic

Total carbon emissions for the NUH campus were lower in 2020 than in 2019 (2019: 75,192 tCO_2_e. 2020: 73,546 tCO_2_e) (Additional Fig. 13a). A comparison of carbon emissions in each month showed no significant differences between 2019 and 2020, except in scope 3 (total carbon emissions, p = 0.18; scope 1, p = 0.95; scope 2, p = 0.13; scope 3, p < 0.0001) (Additional Fig. 13b and c). In scope 3, the amount of solid waste decreased significantly from 2019 to 2020 (46,219.50 [43,784.25, 47,937.50] kg and 42,379.00 [40,210.25, 44,453.25] kg, respectively, p = 0.015) (Table 1). However, we observed no significant difference in the amount of sewage, scrap metal, or non-infectious medical waste, or in the emissions from non-infectious medical waste between 2019 and 2020 (Table 1). The amount of infectious medical waste increased significantly from 2019 to 2020 (44,890.00 [42,332.50, 46,235.00] kg and 57,890.00 [55,120.00, 59,150.00] kg, respectively, p < 0.001) (Table 1).

**Table 1:** Comparison of scope 3 factors between 2019 and 2020

	2019	2020	p-value
**Clean water, (m** ^**3**^ **), median [IQR]**	23,004·50 [21,291·00, 27,745·00]	19,371·00 [18,193·00, 23,871·00]	0·011
**Emissions from clean water, (tCO**_**2**_ **e), median [IQR]**	2·92 [2·70, 3·52]	2·36 [2·22, 2·91]	0·011
**Sewage, (m** ^**3**^ **), median [IQR]**	32,179·50 [29,782·00, 39,306·00]	29,321·00 [28,259·00, 33,654·00]	0·165
**Emissions from sewage, (tCO**_**2**_ **e), median [IQR]**	9·24 [8·55, 11·28]	7·98 [7·69, 9·15]	0·011
**Emissions from medications and medical supplies, (tCO**_**2**_ **e), median [IQR]**	4,019·37 [4,019·37, 4,019·37]	3,940·52 [3,940·52, 3,940·52]	< 0·001
**Solid waste, (kg), median [IQR]**	46,219·50 [43,784·25, 47,937·50]	42,379·00 [40,210·25, 44,453·25]	0·015
**Emissions from solid waste, (tCO**_**2**_ **e), median [IQR]**	37·96 [35·96, 39·38]	34·81 [33·03, 36·51]	0·015
**Scrap metal, (kg), median [IQR]**	440·00 [407·50, 480·00]	400·00 [352·50, 427·50]	0·093
**Infectious medical waste, (kg), median [IQR]**	44,890·00 [42,332·50, 46,235·00]	57,890·00 [55,120·00, 59,150·00]	< 0·001
**Emissions from infectious medical waste, (tCO**_**2**_ **e), median [IQR]**	114·47 [107·95, 117·90]	147·62 [140·56, 150·83]	< 0·001
**Non-infectious medical waste, (kg), median [IQR]**	9,290·00 [9,052·50, 9,955·00]	9,505·00 [8,780·00, 10,595·00]	0·583
**Emissions from non-infectious medical waste, (tCO**_**2**_ **e), median [IQR]**	23·69 [23·08, 25·39]	24·24 [22·39, 27·02]	0·583
**Average temperature, (degree Celsius), median [IQR]**	17·20 [10·22, 23·80]	16·00 [10·88, 24·80]	0·840

IQR: interquartile range; kg: kilogram; tCO_2_e: tonnes of carbon dioxide equivalent

### Sensitivity analysis

Figure 4 shows the Kruskal–Wallis plots of the number of admissions per month, the number of occupied beds per month, the monthly carbon footprint per admission, and the monthly carbon footprint per occupied bed. A significant difference was observed in both the number of admissions per month and the monthly carbon footprint per admission between 2018 and 2020 (Fig. 4a and c) (admission number: 25,907.00 [25,532.50, 26,735.25] in 2018 and 23,534.00 [23,023.00, 24,804.75] in 2020, p = 0.0021; carbon footprint per admission: 0.24 [0.23, 0.24] tCO_2_e in 2018 and 0.26 [0.25, 0.27] eCO_2_e in 2020, p = 0.001). Notably, although the number of admissions per month decreased, the monthly carbon footprint increased. Conversely, we observed no significant difference in either the number of occupied beds per month or the monthly carbon footprint per occupied bed (Fig. 4b and d) (p = 1.00 and p = 0.18, respectively).

### Hospital data

The annual number of admissions exhibited a general decrease over the study period (Additional Fig. 14). Moreover, the total monthly number of outpatients in 2020 was typically lower than that in other years (Additional Fig. 15). Although the length of the average hospital stay typically decreased over the study period (Additional Fig. 16), the average hospital stay increased in 2020 (12.2 days). Finally, the number of occupied beds per year decreased throughout the study period, except in 2015, then decreased in 2020 by almost 13% from the peak value in 2015 (Additional Fig. 17).

## Discussion

The results of this study show that the carbon footprint of the NUH campus has increased over the past 10 years, primarily owing to the rising use of pharmaceuticals and medical supplies. Overall, monthly carbon emissions were positively associated with the monthly average temperature. The amount of solid waste decreased significantly during the COVID-19 pandemic, whereas the amount of infectious medical waste increased from pre-COVID-19 levels. As the variables used in this study are relatively easy to obtain and the emission factors are published by either local government or the GHG protocol, this study is highly replicable.

Although healthcare services represent a major contribution to total carbon emissions [3], the carbon footprints of individual hospitals remain unclear. Previous studies have reported carbon emissions from medical settings, including dialysis, operation rooms, an army hospital, and remote teleclinics [23–25, 36]. However, to our knowledge, this is the first study to report the overall carbon emissions of a large medical facility over a 10-year period. As NUH treats patients with advanced medical care, it tends to spend more money on pharmaceuticals and medical supplies. Moreover, electricity usage has increased since the opening of a new clinical building. Thus, total emissions have increased over the last 10 years. Introducing renewable energy such as solar panels in a hospital can reduce scope-2 emissions, which account for over 25% to the total carbon footprint of the hospital. Emission factors are defined by the quantity of fuel burned while generating energy. Over the years, Japan has increased its use of renewable energy sources for electricity production and resumed nuclear power plant operations [37]. Notably, in the NUH campus, the carbon footprints of factors other than pharmaceuticals and medical supplies have reduced over the years. Generally, it is difficult to reduce the amount of medicine for patient care, especially considering the ageing population in Japan. However, it is important to consider what is strictly necessary for patient care under increasing scope-3 emissions, particularly with regard to pharmaceuticals and medical supplies. Moreover, the results showed that average external temperature was positively associated with an increased carbon footprint at the NUH campus. This is because electricity and gas consumption are increased to maintain a comfortable indoor temperature, implying that the carbon footprint is likely to increase with the increasing effects of climate change, leading to a vicious circle of temperature rise.

Regarding the impact of COVID-19, we observed a reduction of the overall carbon footprint from 2019 to 2020, which is attributed to the effects of the COVID-19 epidemic in the Nagoya area. Compared with 2019, the amount of pharmaceuticals, water usage, and non-medical waste all decreased in 2020. These reductions are consistent with the reduced number of both outpatients and admissions, which was observed in other hospitals in Japan during the COVID-19 pandemic [38, 39]. Relatively few studies have investigated the effect of COVID-19 on carbon emissions in the healthcare sector. For example, Filimonau et al. reported a reduction in the amount of waste from a university during the COVID-19 pandemic [40]. These results were similar to those of our study because the number of both patients and students declined because of confinement measures. This also led to a decrease in carbon emissions from pharmaceuticals and medical supplies in 2020. However, the amount of infectious medical waste increased rapidly in 2020 because of the increased use of PPE. Indeed, public health measures such as infection control and vaccination are important for human safety and for reducing carbon footprints in medical settings [41, 42]. For example, the carbon footprint of COVID-19 mRNA vaccines was approximately 0.01–0.20 kgCO_2_e in Germany [42]. Vaccines are both highly effective and environmentally friendly compared to the long-term treatment of patients. The observation that the number of patients decreased because of confinement measured during the COVID-19 pandemic suggests that some hospital use may be unnecessary, which could create an environmental burden through unnecessary scope-3 carbon emissions. Such unnecessary hospital use may be explained by the fact that Japan has one of the highest numbers of beds per capita among countries in the Organization for Economic Co-operation and Development [43]. Thus, selecting necessary care for patients is more important than ever.

Despite the reduction in the overall carbon footprint from 2019 to 2020, the overall carbon footprint in 2020 was close to that in 2018. Considering similar operation of NUH from 2018 to 2020, we propose the following reasons for this result. First, the monthly carbon emissions per admission have been increasing with time at NUH, which can be explained by the ageing population in Japan. The older the patient, the more complications they typically have [44]; therefore, more medical care and treatment are required. Second, although the number of admissions decreased in 2020, the average hospital stay (12.2 days) was longer than that in 2018 (11.9 days). This also reflects the need for increased medical care. However, the monthly carbon emissions per occupied bed did not change, which suggests a change in patient characteristics rather than a systematic change in NUH. Third, carbon emissions from pharmaceuticals were higher in 2020 than in 2018, despite the lower number of admissions; this is attributed to the COVID-19 pandemic in Japan, which increased the number of patients requiring more intensive medical care. As stated above, more patients required more intensive medical care at NUH in 2020. Furthermore, NUH cares for patients with complex backgrounds such as refractory cancers, who require more expensive medications, leading to increased pharmaceutical bills. Therefore, because the carbon footprint per admission has increased because of the COVID-19 pandemic and an ageing society, public health monitoring should be urgently conducted to reduce the need for medical care and avoid associated complications.

This study is a longitudinal case study of the carbon footprint at a large medical facility over the last 10 years, which indicates that the overall carbon footprint is increasing. Because the variables used in the study are relatively easy to obtain, we suggest that hospitals start publishing their overall carbon footprint data to allow more research of this type. Moreover, this study also shows that the carbon footprint per admission is increasing. This trend is likely to be similar in other countries with ageing populations; therefore, we suggest that countries worldwide place an emphasis on public health monitoring.

This study has the following limitations. First, this study was based on a single healthcare facility. Nevertheless, we effectively capture the trends in a large medical facility over a 10-year period. Second, it was not possible to accurately determine scope-3 emissions. For example, we could not gain data regarding commutes of hospital staff, students, and patients. However, as the variables in this study are relatively easy to acquire in other hospitals, at least in Japan, other hospitals should be able to replicate the protocol. Third, the overall carbon footprint of NUH encompasses clinical work, research, and education. Therefore, although the carbon footprint per admission and occupied bed were used for the sensitivity analyses in this study, they do not represent the true value because carbon emissions from education, outpatients, and research were not included.

## Conclusion

This 10-year longitudinal study showed that the overall carbon footprint at NUH has increased over the last decade and was impacted by the COVID-19 pandemic. Moreover, the COVID-19 impact highlighted the possibility of unnecessary hospital use and increasing patient care demands. Therefore, accurate monitoring and evaluation of carbon emissions in the medical sector are urgently required around the world to enable effective action against the climate crisis. Future work should perform carbon footprint analyses of multiple healthcare centres to determine accurate carbon footprints for the healthcare industry. Additionally, researchers should perform detailed analyses of the carbon footprint of COVID-19-related medical care.

## Supplementary Information

### Electronic Supplementary Material

Below is the link to the electronic supplementary material.

Supplementary Material 1

### Electronic Supplementary Material

Below is the link to the electronic supplementary material.

Supplementary Material 2

## Data Availability

All data generated or analysed during this study are included in this published article [and its supplementary information files].

## Declarations

### Additional files

Additional File 1 (pdf): Additional Figures and Tables.

Additional File 2 (xlsx): Original raw data.

## Abbreviations

COVID-19: Coronavirus disease 2019
PPE: personal protective equipment
NUH: Nagoya University Hospital
