# Supplementary material for: Influence of COVID-19 on the 10-year carbon footprint of the Nagoya University Hospital and medical research centre
Source: Global Health. 2022 Nov 7;18:92. doi: 10.1186/s12992-022-00883-9 (PMC9640843; doi:10.1186/s12992-022-00883-9)
Supplement: Supplementary file 1 — Supplementary Material 1 [file 12992_2022_883_MOESM1_ESM.pdf]

Additional File 1

Additional Table 1. Emission factors for each year from 2010 to 2020.

	2010	2011	2012	2013	2014	2015	2016	2017	2018	2019	2020
**Electricity (tCO_2e/kWh)**	0·000473	0·000518	0·000516	0·000513	0·000497	0·000486	0·000485	0·000476	0·000458	0·000431	0·000406
**Gas (tCO_2e/Nm^3)**	0·000223	0· 000223	0· 000223	0·000223	0· 000223	0·000223	0·000223	0·000223	0·000223	0·000223	0·000223
**Clean water (tCO_2e/m^3)**	0·00013	0·000147	0·000146	0·000143	0·000139	0·000135	0·000137	0·000135	0·000132	0·000127	0·000122
**Sewage (tCO_2e/m^3)**	0·000293	0·000302	0·000287	0·000307	0·000291	0·00027	0·000302	0·000292	0·000295	0·000287	0·000272
**Solid waste (tCO_2e/kg)**	NA	NA	NA	NA	0·0008214	0·0008214	0·0008214	0·0008214	0·0008214	0·0008214	0·0008214
**Scrap metal (tCO_2e/kg)**	NA	NA	NA	NA	0·0000122	0·0000122	0·0000122	0·0000122	0·0000122	0·0000122	0·0000122
**Medical waste (infectious) (tCO_2e/kg)**	NA	NA	NA	NA	0·00255	0·00255	0·00255	0·00255	0·00255	0·00255	0·00255
**Medical waste (non-infectious) (tCO_2e/kg)**	NA	NA	NA	NA	0·00255	0·00255	0·00255	0·00255	0·00255	0·00255	0·00255
**Pharmaceuticals (tCO_2e/Yen)**	0·00000283	0·00000283	0·00000283	0·00000283	0·00000283	0·00000283	0·00000283	0·00000283	0·00000283	0·00000283	0·00000283
**Medical supplies (tCO_2e/Yen)**	0·00000329	0·00000329	0·00000329	0·00000329	0·00000329	0·00000329	0·00000329	0·00000329	0·00000329	0·00000329	0·00000329

Note: kg: kilogram; kWh: kilowatt; NA: Not available; tCO_2e: tonnes of carbon dioxide equivalent.

References for emission factors:

Electricity: The Chubu Electric Power's website (https://miraiz.chuden.co.jp/info/topics/__icsFiles/afieldfile/2020/09/16/topics_att_20200916_01.pdf.pdf). For an emission factor in 2020, (https://miraiz.chuden.co.jp/info/topics/1206545_1939.html).

Gas: https://ghg-santeikohyo.env.go.jp/files/calc/itiran_2020_rev.pdf

Clean water and sewage: Answered by the Nagoya City Waterworks & Sewerage Bureau.

Solid waste, scrap metal, pharmaceuticals, and medical supplies: https://www.env.go.jp/earth/ondanka/supply_chain/gvc/estimate_tool.html#no07

Medical waste (both infectious and non-infectious): https://www.env.go.jp/earth/ondanka/suishin_g/3rd_edition/ref2.pdf

Additional Table 2. Carbon emission sources for each year between 2010 and 2020 (values are the median of 12 monthly values in a year, with the interquartile range in parentheses).

	2010	2011	2012	2013	2014
**Gas, (m^3)**	189,142·00 [174,571·00, 231,952·25]	182,888·50 [145,141·00, 209,253·25]	178,008·00 [153,299·75, 194,791·50]	172,378·50 [150,541·25, 207,928·25]	174,292·50 [141,698·50, 200,211·50]
**Emission from gas, (tCO2e)**	421·79 [389·29, 517·25]	407·84 [323·66, 466·63]	396·96 [341·86, 434·39]	384·40 [335·71, 463·68]	388·67 [315·99, 446·47]
**Electricity, (kWh)**	3,544,415·50 [3,391,485·00, 4,054,412·00]	3,435,184·50 [3,331,413·50, 3,890,282·50]	3,470,566·00 [3,287,747·00, 3,719,727·25]	3,526,377·00 [3,365,848·00, 3,889,756·25]	3,577,543·50 [3,459,199·00, 3,924,728·75]
**Emission from electricity, (tCO2e)**	1,676·51 [1,604·17, 1,917·74]	1,779·43 [1,725·67, 2,015·17]	1,790·81 [1,696·48, 1,919·38]	1,809·03 [1,726·68, 1,995·44]	1,778·04 [1,719·22, 1,950·59]
**Clean water, (m^3)**	23,397·00 [21,923·00, 24,667·00]	23,414·00 [20,368·00, 26,292·00]	21,208·00 [19,331·00, 25,248·00]	19,303·50 [18,011·00, 23,696·00]	17,901·50 [17,326·00, 23,583·00]
**Emission from clean water, (tCO_2e)**	3·04 [2·85, 3·21]	3·44 [2·99, 3·86]	3·10 [2·82, 3·69]	2·76 [2·58, 3·39]	2·49 [2·41, 3·28]
**Sewage, (m^3)**	39,860·00 [35,341·00, 42,226·00]	36,087·00 [33,532·00, 39,230·00]	31,735·00 [28,453·00, 35,137·00]	30,904·50 [29,410·00, 34,536·00]	29,222·50 [28,728·00, 35,585·00]
**Emission from sewage, (tCO_2e)**	11·68 [10·35, 12·37]	10·90 [10·13, 11·85]	9·11 [8·17, 10·08]	9·49 [9·03, 10·60]	8·50 [8·36, 10·36]
**Emission from medicine, (tCO_2e)**	2,437·40 [2,437·40, 2,437·40]	2,798·95 [2,798·95, 2,798·95]	3,018·22 [3,018·22, 3,018·22]	3,307·55 [3,307·55, 3,307·55]	3,528·61 [3,528·61, 3,528·61]
**Solid waste, (kg)**	NA	NA	NA	NA	46,976·00 [44,608·75, 48,179·00]
**Emission from solid waste, (tCO_2e)**	NA	NA	NA	NA	38·59 [36·64, 39·57]
**Scrap metal, (kg)**	NA	NA	NA	NA	530·00 [520·00, 620·00]
**Emission from scrap metal, (tCO_2e)**	NA	NA	NA	NA	0·01 [0·01, 0·01]
**Medical waste (infectious), (kg)**	NA	NA	NA	NA	38,334·00 [36,829·75, 39,703·75]
**Emission from medical waste (infectious), (tCO_2e)**	NA	NA	NA	NA	97·75 [93·92, 101·24]
**Medical waste (non-infectious), (kg)**	NA	NA	NA	NA	11,660·00 [11,087·50, 12,110·00]
**Emission from medical waste (non-infectious), (tCO_2e)**	NA	NA	NA	NA	29·73 [28·27, 30·88]
**Scope 1, (tCO_2e)**	421·79 [389·29, 517·25]	407·84 [323·66, 466·63]	396·96 [341·86, 434·39]	384·40 [335·71, 463·68]	388·67 [315·99, 446·47]
**Scope 2, (tCO_2e)**	1,676·51 [1,604·17, 1,917·74]	1,779·43 [1,725·67, 2,015·17]	1,790·81 [1,696·48, 1,919·38]	1,809·03 [1,726·68, 1,995·44]	1,778·04 [1,719·22, 1,950·59]

**Scope 3 without waste, (tCO_2e)**	2,452·09 [2,450·66, 2,452·98]	2,813·17 [2,812·07, 2,814·61]	3,030·42 [3,029·20, 3,031·99]	3,319·70 [3,319·10, 3,321·54]	3,539·58 [3,539·40, 3,542·24]
**Scope 3 with waste, (tCO_2e)**	NA	NA	NA	NA	3704·60 [3701·06, 3711·31]
**Emission without waste, (tCO_2e)**	4,571·77 [4,498·01, 4,833·19]	5,066·30 [4,924·75, 5,203·18]	5,208·93 [5,162·56, 5,442·37]	5,547·94 [5,471·65, 5,718·49]	5,729·66 [5,626·97, 5,849·74]
**Emission with waste, (tCO_2e)**	NA	NA	NA	NA	5,910·61 [5,781·36, 6,011·71]
**Average monthly temperature, (℃)**	16·00 [7·80, 24·45]	16·35 [7·90, 24·12]	16·60 [9·20, 23·18]	16·60 [8·58, 23·93]	16·75 [8·70, 23·55]

	**2015**	**2016**	**2017**	**2018**	**2019**	**2020**	**p**
**Gas, (m^3)**	159,791·00 [129,491·75, 184,504·00]	171,611·50 [157,966·75, 206,460·75]	155,943·50 [131,526·50, 205,045·25]	173,122·00 [148,836·25, 211,692·75]	164,967·00 [129,572·50, 191,875·25]	163,675·50 [132,427·00, 188,383·75]	0·570
**Emission from gas, (tCO_2e)**	356·33 [288·77, 411·44]	382·69 [352·27, 460·41]	347·75 [293·30, 457·25]	386·06 [331·90, 472·07]	367·88 [288·95, 427·88]	365·00 [295·31, 420·10]	0·570
**Electricity, (kWh)**	3,546,714·50 [3,428,474·75, 3,861,134·00]	3,605,080·00 [3,449,287·00, 3,966,512·75]	3,767,851·50 [3,664,384·00, 3,941,978·75]	3,811,958·50 [3,637,235·75, 4,115,118·50]	3,760,401·50 [3,638,998·50, 4,187,604·25]	3,832,045·00 [3,634,811·25, 4,206,047·50]	0·178
**Emission from electricity, (tCO_2e)**	1,723·70 [1,666·24, 1,876·51]	1,748·46 [1,672·90, 1,923·76]	1,793·50 [1,744·25, 1,876·38]	1,745·88 [1,665·85, 1,884·72]	1,620·73 [1,568·41, 1,804·86]	1,555·81 [1,475·73, 1,707·66]	0·024
**Clean water, (m^3)]**	19,897·00 [19,130·00, 23,140·00]	20,132·50 [17,819·00, 22,319·00]	19,130·00 [18,823·00, 22,796·00]	22,288·50 [21,042·00, 24,970·00]	23,004·50 [21,291·00, 27,745·00]	19,371·00 [18,193·00, 23,871·00]	0·001
**Emission from clean water, (tCO_2e)**	2·69 [2·58, 3·12]	2·76 [2·44, 3·06]	2·58 [2·54, 3·08]	2·94 [2·78, 3·30]	2·92 [2·70, 3·52]	2·36 [2·22, 2·91]	< 0·001
**Sewage, (m^3)**	31,622·00 [29,614·00, 35,142·00]	32,511·50 [28,628·00, 34,442·00]	31,313·50 [30,116·00, 34,361·00]	33,817·50 [32,674·00, 38,376·00]	32,179·50 [29,782·00, 39,306·00]	29,321·00 [28,259·00, 33,654·00]	< 0·001
**Emission from sewage, (tCO_2e)**	8·54 [8·00, 9·49]	9·82 [8·65, 10·40]	9·14 [8·79, 10·03]	9·98 [9·64, 11·32]	9·24 [8·55, 11·28]	7·98 [7·69, 9·15]	< 0·001
**Emission from medicine, (tCO_2e)**	3,592·69 [3,592·69, 3,592·69]	3,604·80 [3,604·80, 3,604·80]	3,637·42 [3,637·42, 3,637·42]	3,743·86 [3,743·86, 3,743·86]	4,019·37 [4,019·37, 4,019·37]	3,940·52 [3,940·52, 3,940·52]	< 0·001
**Solid waste, (kg)**	45,878·50 [43,419·50, 47,368·00]	46,044·00 [44,653·25, 46,718·50]	46,321·50 [44,247·00, 47,387·75]	47,409·00 [46,490·25, 48,832·00]	46,219·50 [43,784·25, 47,937·50]	42,379·00 [40,210·25, 44,453·25]	0·011
**Emission from solid waste, (tCO_2e)**	37·68 [35·66, 38·91]	37·82 [36·68, 38·37]	38·05 [36·34, 38·92]	38·94 [38·19, 40·11]	37·96 [35·96, 39·38]	34·81 [33·03, 36·51]	0·011

**Scrap metal, (kg)**	540·00 [497·50, 652·50]	495·00 [475·00, 560·00]	520·00 [460·00, 580·00]	470·00 [440·00, 530·00]	440·00 [407·50, 480·00]	400·00 [352·50, 427·50]	< 0·001
**Emission from scrap metal, (tCO_2e)**	0·01 [0·01, 0·01]	0·01 [0·01, 0·01]	0·01 [0·01, 0·01]	0·01 [0·01, 0·01]	0·01 [0·00, 0·01]	0·00 [0·00, 0·01]	< 0·001
**Medical waste (infectious), (kg)**	41,742·50 [39,682·75, 43,595·75]	41,178·50 [40,695·00, 42,960·50]	41,280·00 [40,495·00, 42,717·50]	43,665·00 [42,175·00, 45,977·50]	44,890·00 [42,332·50, 46,235·00]	57,890·00 [55,120·00, 59,150·00]	< 0·001
**Emission from medical waste (infectious), (tCO_2e)**	106·44 [101·19, 111·17]	105·01 [103·77, 109·55]	105·26 [103·26, 108·93]	111·35 [107·55, 117·24]	114·47 [107·95, 117·90]	147·62 [140·56, 150·83]	< 0·001
**Medical waste (non-infectious), (kg)**	11,457·00 [10,930·00, 12,002·50]	10,690·00 [10,235·00, 10,862·50]	10,195·00 [9,420·00, 10,472·50]	10,825·00 [10,510·00, 11,070·00]	9,290·00 [9,052·50, 9,955·00]	9,505·00 [8,780·00, 10,595·00]	< 0·001
**Emission from medical waste (non-infectious), (tCO_2e)**	29·22 [27·87, 30·61]	27·26 [26·10, 27·70]	26·00 [24·02, 26·70]	27·60 [26·80, 28·23]	23·69 [23·08, 25·39]	24·24 [22·39, 27·02]	< 0·001
**Scope 1, (tCO_2e)**	356·33 [288·77, 411·44]	382·69 [352·27, 460·41]	347·75 [293·30, 457·25]	386·06 [331·90, 472·07]	367·88 [288·95, 427·88]	365·00 [295·31, 420·10]	0·570
**Scope 2, (tCO_2e)**	1,723·70 [1,666·24, 1,876·51]	1,748·46 [1,672·90, 1,923·76]	1,793·50 [1,744·25, 1,876·38]	1,745·88 [1,665·85, 1,884·72]	1,620·73 [1,568·41, 1,804·86]	1,555·81 [1,475·73, 1,707·66]	0·024
**Scope 3 without waste, (tCO_2e)**	3,603·91 [3,603·27, 3,605·30]	3,617·38 [3,615·84, 3,618·26]	3,649·11 [3,648·77, 3,650·53]	3,756·78 [3,756·15, 3,758·48]	4,031·52 [4,030·64, 4,034·17]	3,950·80 [3,950·53, 3,952·58]	< 0·001
**Scope 3 with waste, (tCO_2e)**	3,779·23 [3,768·46, 3,786·33]	3,789·57 [3,785·05, 3,791·15]	3,817·30 [3,814·88, 3,825·85]	3,936·35 [3,926·97, 3,942·05]	4,206·52 [4,200·88, 4,214·15]	4,157·07 [4,154·17, 4,160·05]	< 0·001
**Emission without waste, (tCO_2e)**	5,714·69 [5,654·98, 5,796·50]	5,756·48 [5,693·77, 5,928·51]	5,838·56 [5,724·26, 5,933·23]	5,885·67 [5,778·91, 6,017·09]	6,029·14 [5,971·20, 6,128·39]	5,855·63 [5,774·43, 6,082·18]	< 0·001
**Emission with waste, (tCO_2e)**	5,889·88 [5,826·59, 5,968·39]	5,924·88 [5,862·87, 6,099·16]	6,010·07 [5,892·79, 6,103·71]	6,061·99 [5,962·84, 6,192·01]	6,209·19 [6,139·21, 6,294·47]	6,057·13 [5,975·74, 6,277·47]	0·006
**Average monthly temperature, (℃)**	16·80 [10·20, 22·50]	17·80 [8·33, 23·47]	16·30 [9·82, 22·70]	17·70 [9·60, 23·45]	17·20 [10·22, 23·80]	16·00 [10·88, 24·80]	1·000

Note: kg: kilogram; kWh: kilowatt; NA: Not available; tCO_2e: tonnes of carbon dioxide equivalent; p: p-value

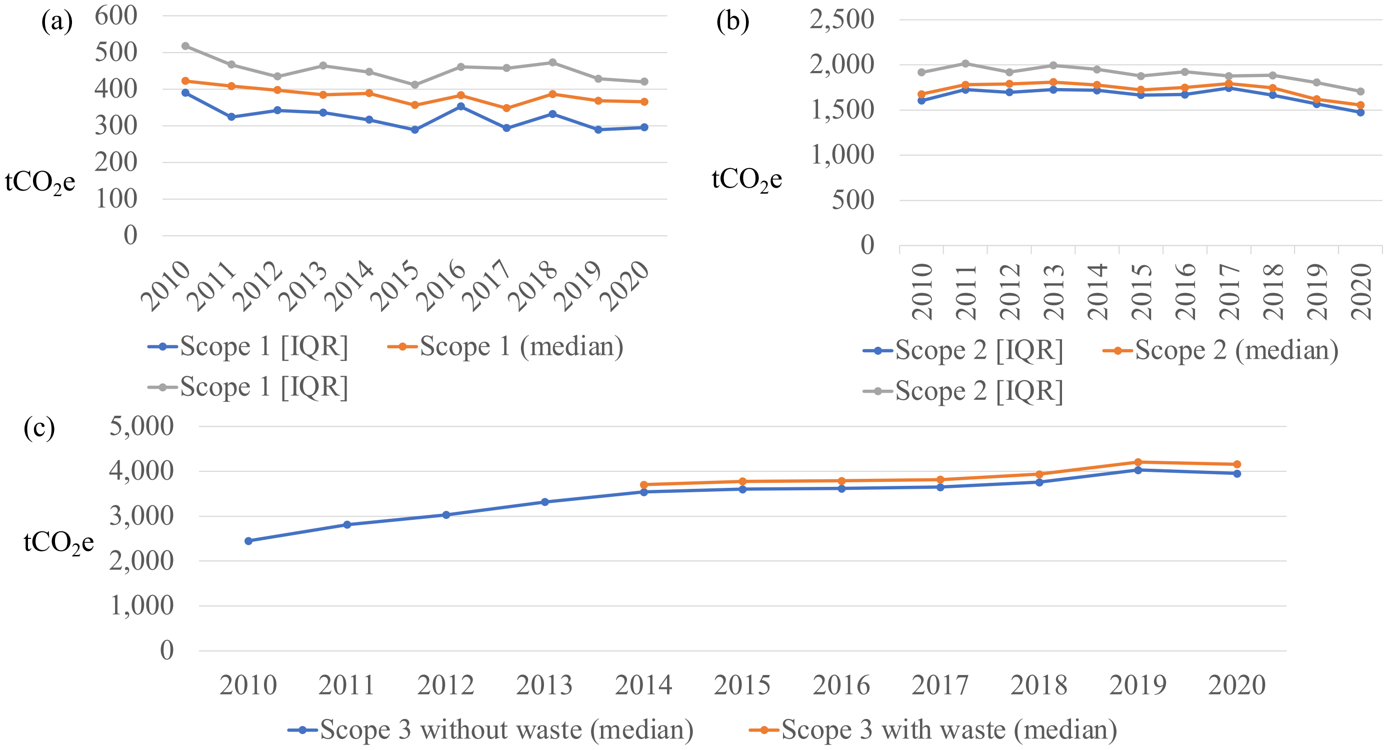

Additional Figure 1. Monthly carbon footprint of each emissions scope from 2010 to 2020. (a) Scope 1; (b) scope 2; (c) scope 3 with and without waste. Because the interquartile ranges were too close to the median, only the median values are shown.

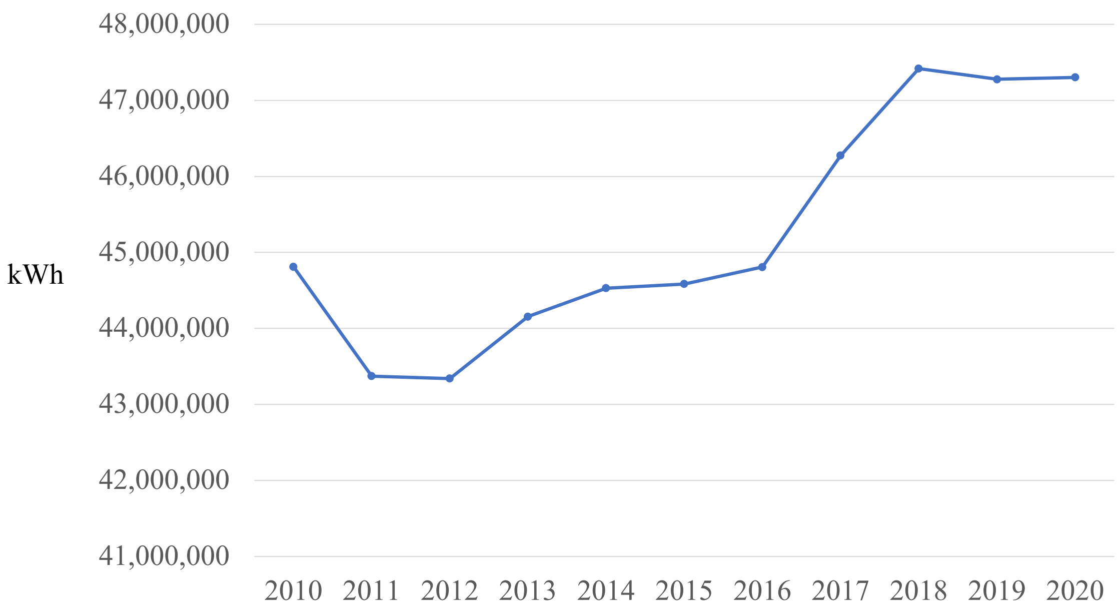

Additional Figure 2. Electricity usage from 2010 to 2020.

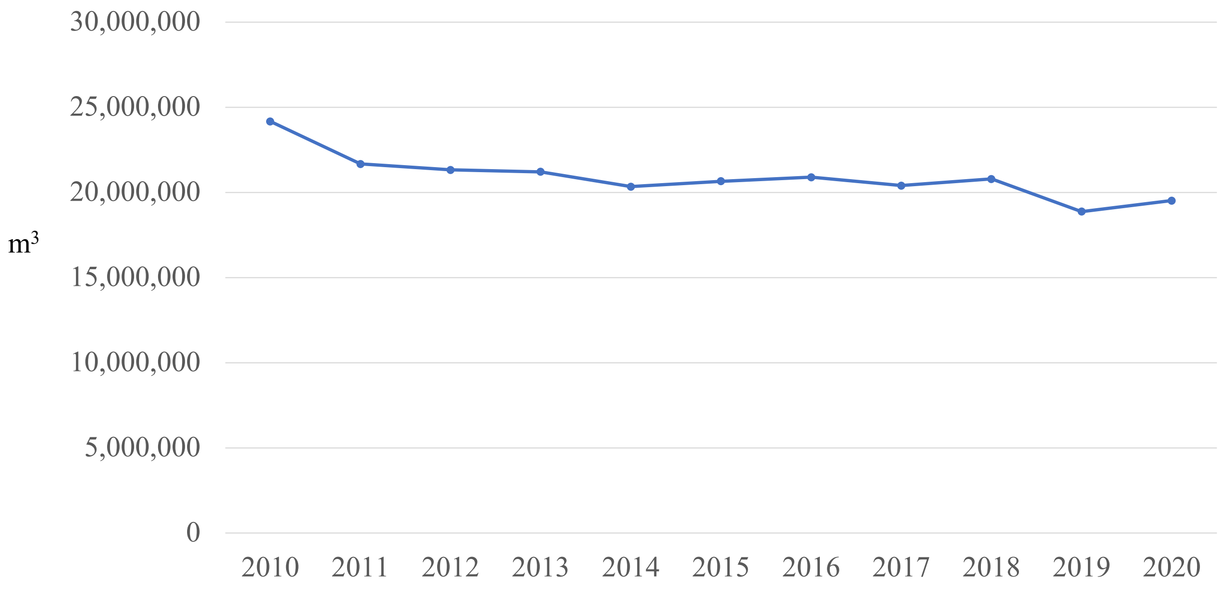

Additional Figure 3. Gas usage from 2010 to 2020.

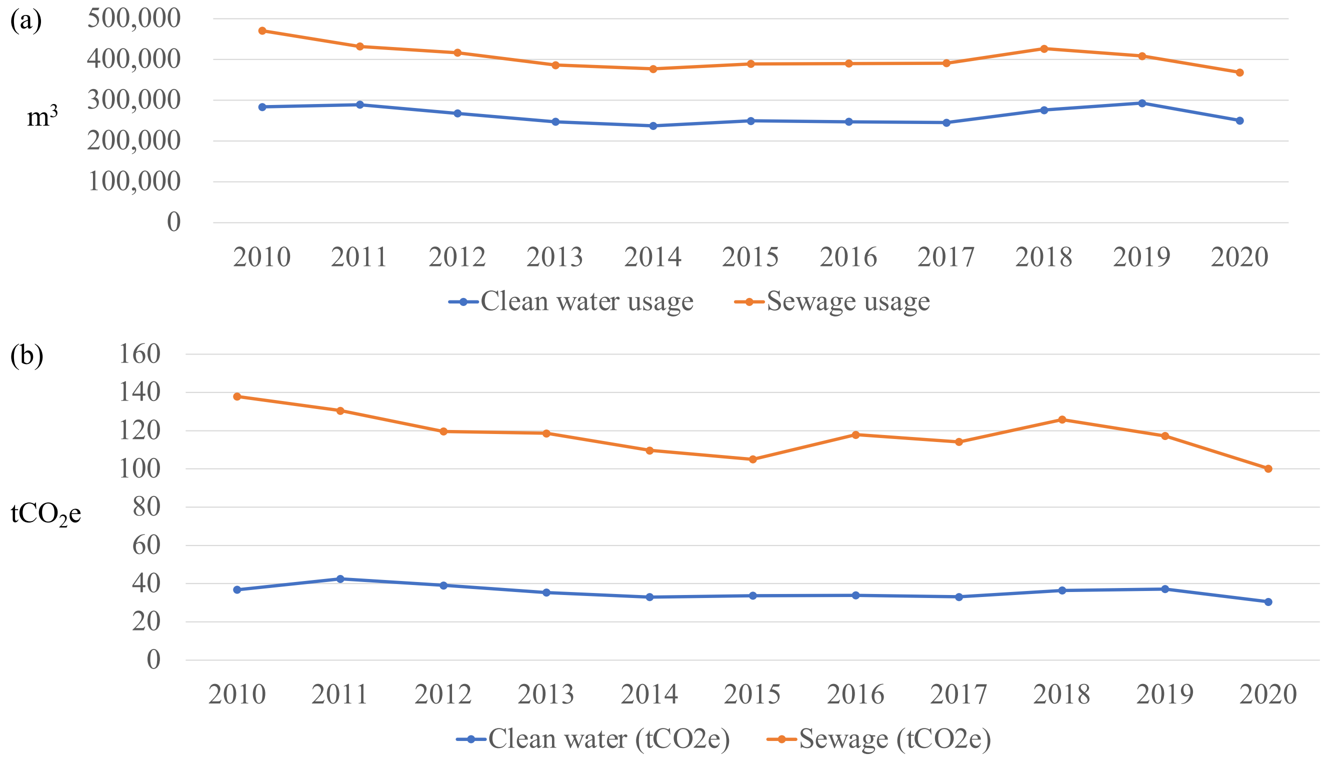

Additional Figure 4. Annual usage and carbon footprint of clean water and sewage from 2010 to 2020: (a) usage; (b) carbon footprint

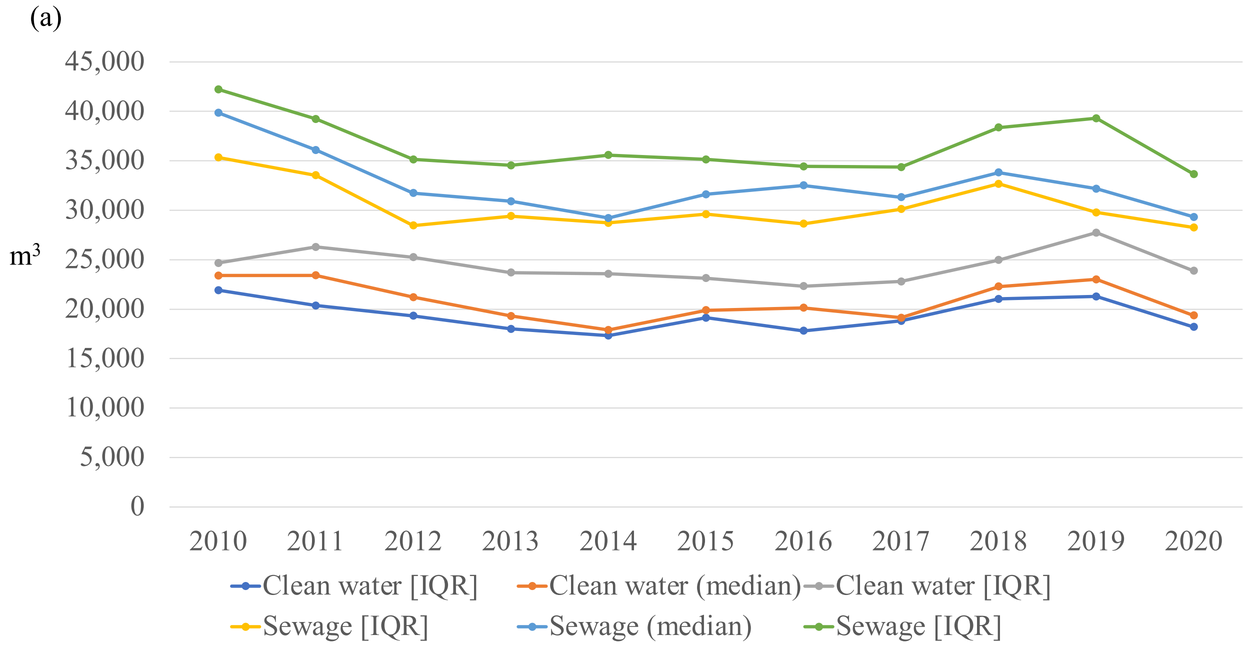

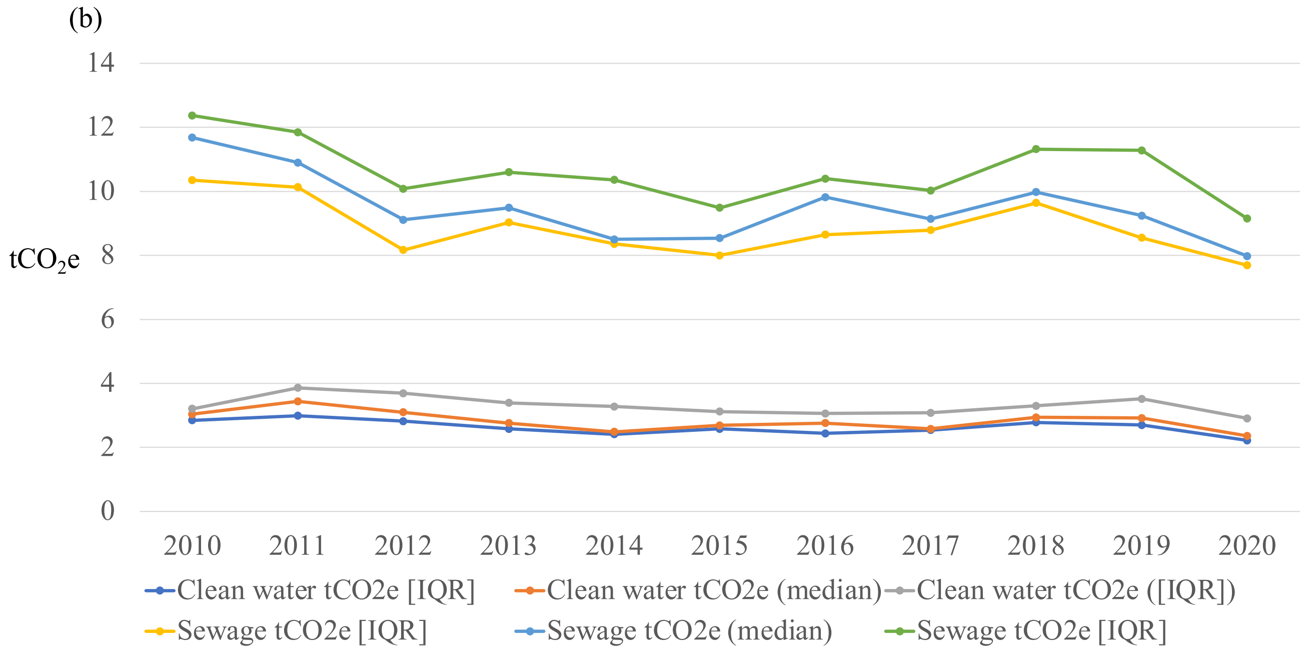

Additional Figure 5. Monthly usage and carbon footprint of clean water and sewage from 2010 to 2020: (a) usage; (b) carbon footprint.

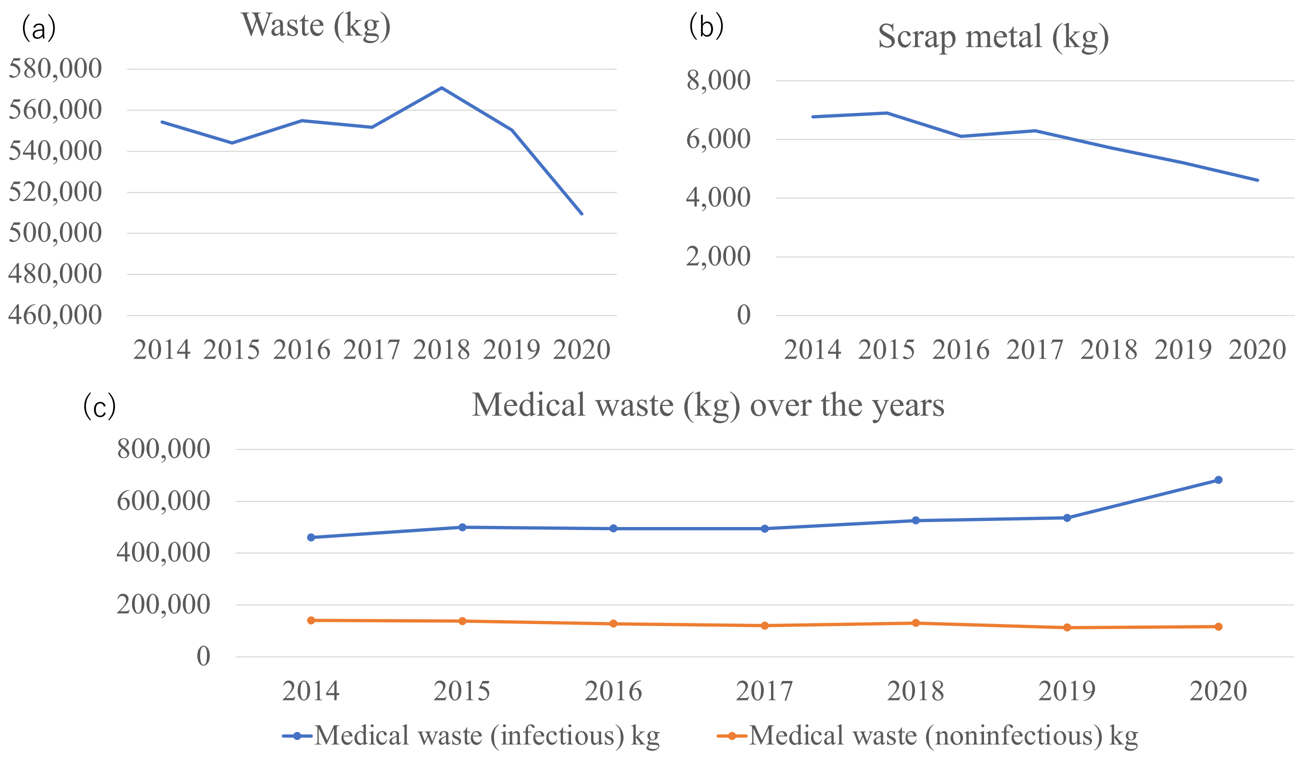

Additional Figure 6. Amount of waste (kg) from 2010 to 2020: (a) solid waste; (b) scrap waste; (c) medical waste.

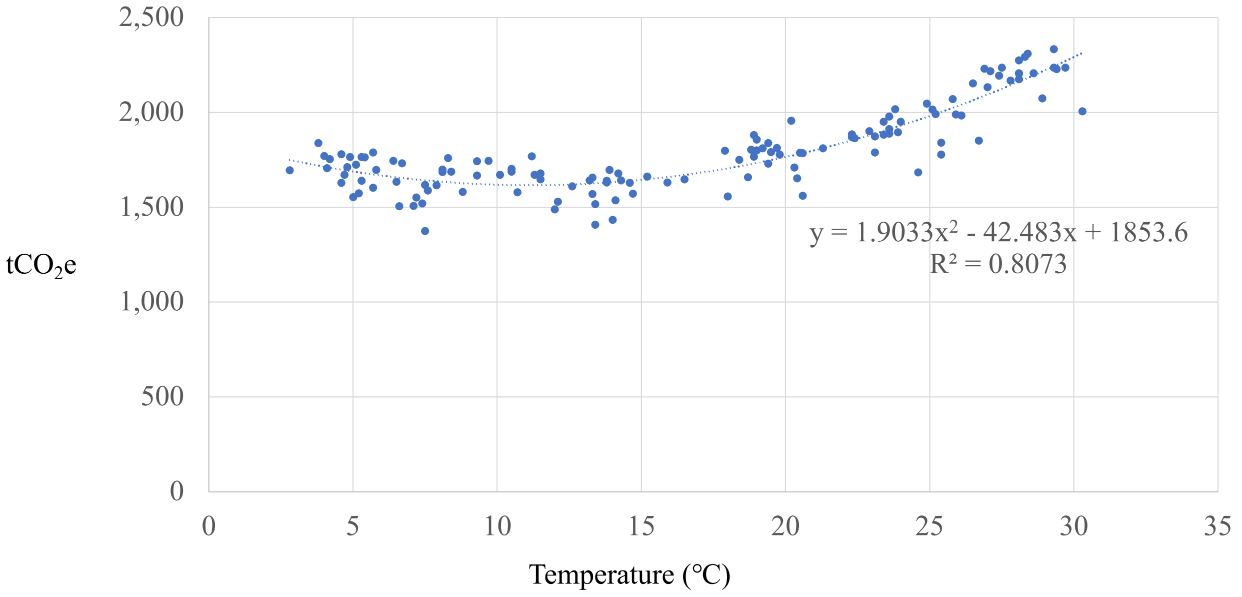

Additional Figure 7. Scatter plot and trendline showing the relationship between electricity usage (kWh) and monthly average external temperature from April 2014 to March 2021.

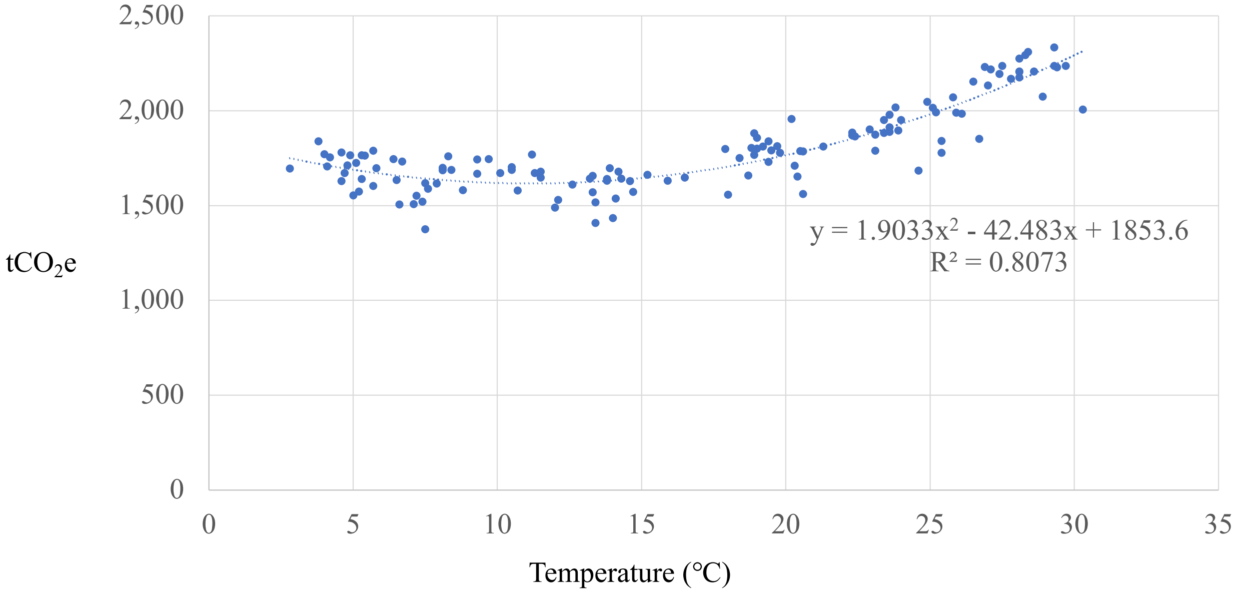

Additional Figure 8. Scatter plot and trendline showing the relationship between carbon emissions from electricity usage (tCO_2e) and monthly average external temperature from April 2010 to March 2021.

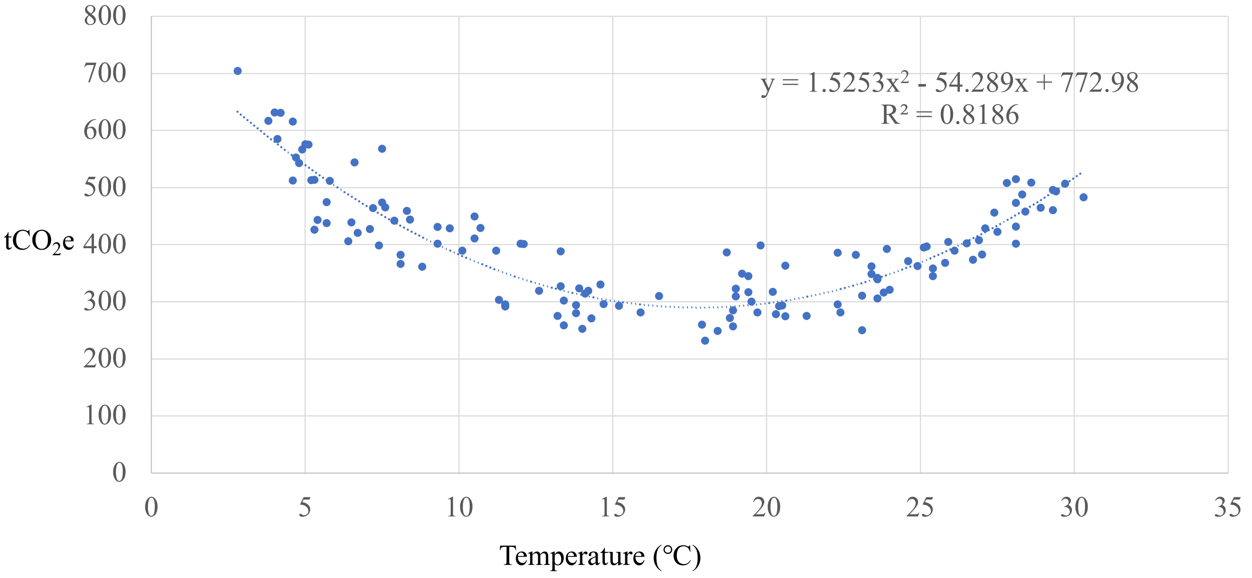

Additional Figure 9. Scatter plot and trendline showing the relationship between carbon emissions from gas usage (tCO_2e) and monthly average external temperature from April 2010 to March 2021.

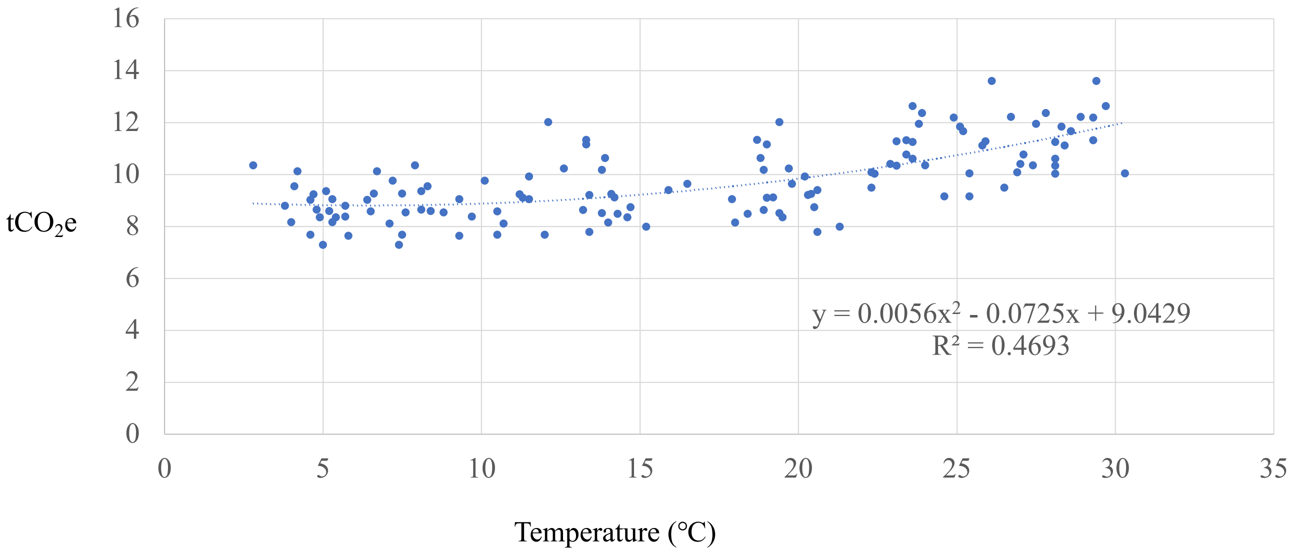

Additional Figure 10. Scatter plot and trendline showing the relationship between carbon emissions from sewage usage (tCO_2e) and monthly average external temperature from April 2010 to March 2021.

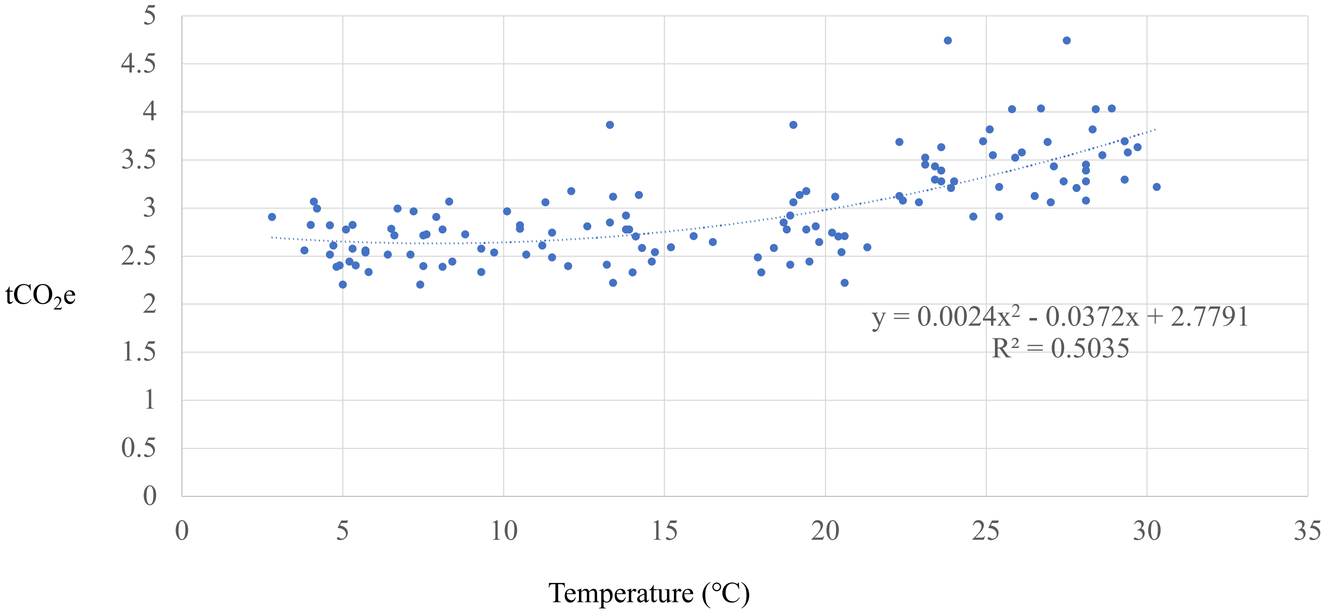

Additional Figure 11. Scatter plot and trendline showing the relationship between carbon emissions from clean water usage (tCO_2e) and monthly average external temperature from April 2010 to March 2021.

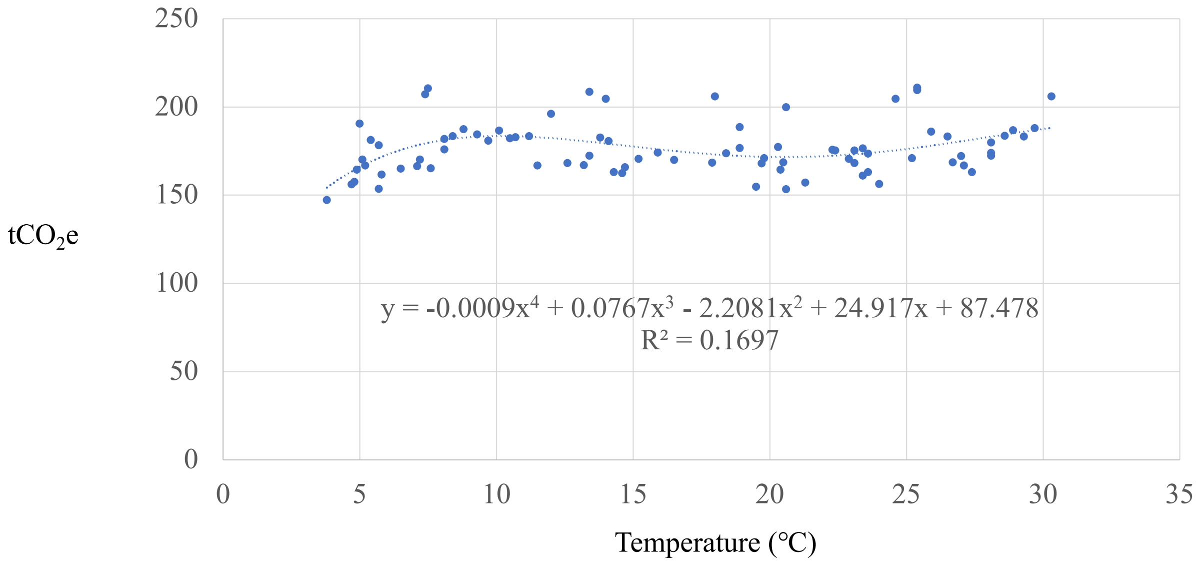

Additional Figure 12. Scatter plot and trendline showing the relationship between carbon emissions from waste (tCO2e) and temperature from April 2014 to March 2021.

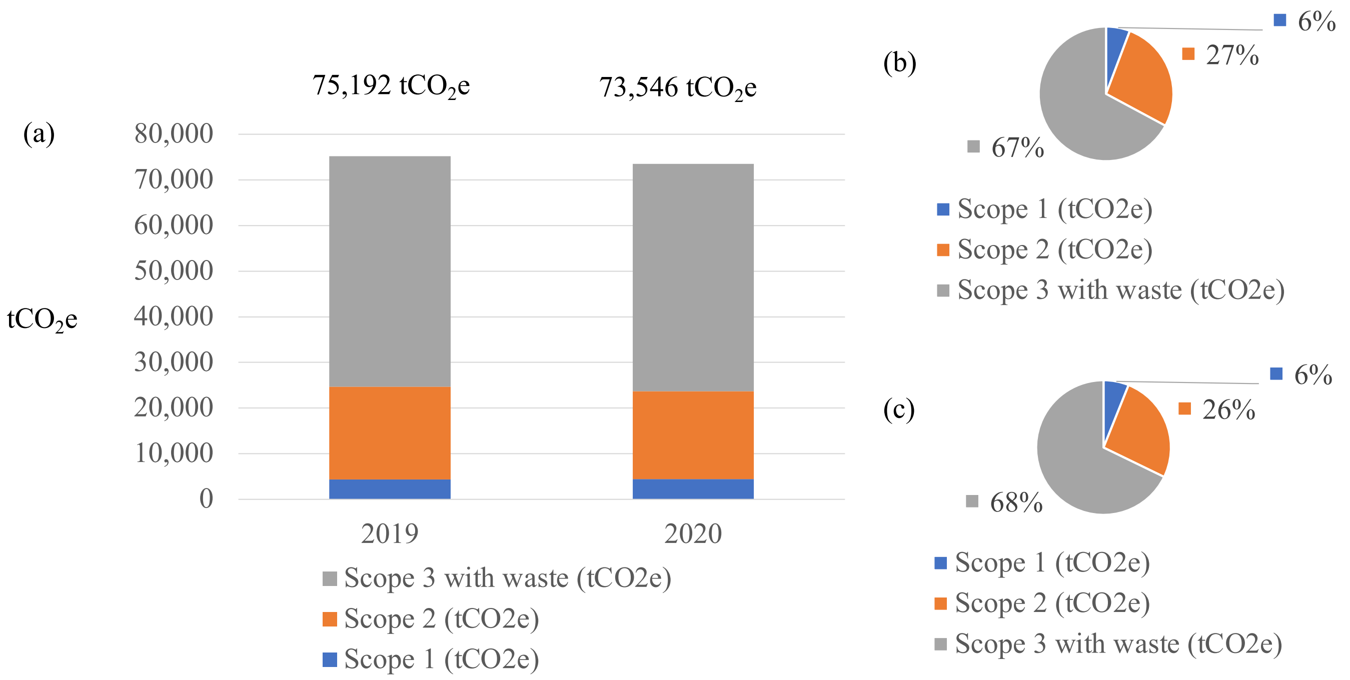

Additional Figure 13. (a) Total carbon footprint and (b–c) relative proportions of each emission scope for 2019 and 2020.

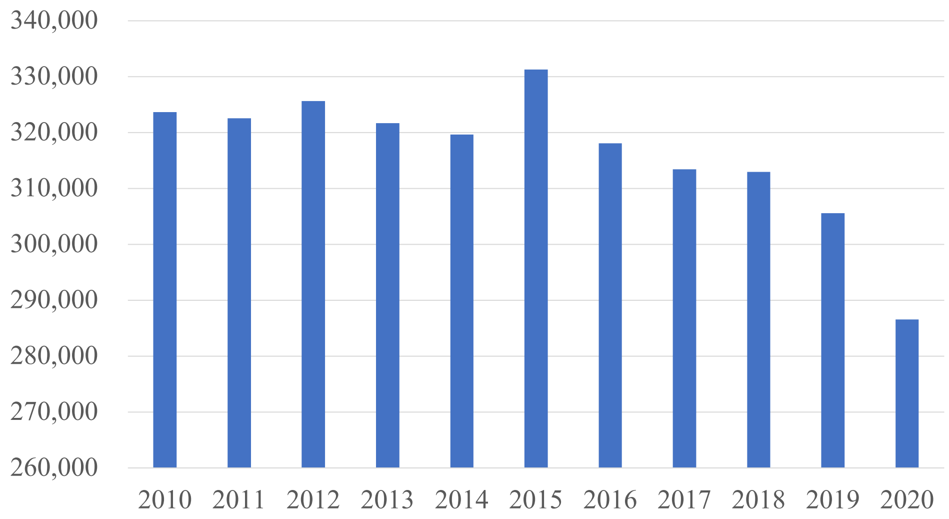

Additional Figure 14. Total annual inpatient admissions from 2010 to 2020.

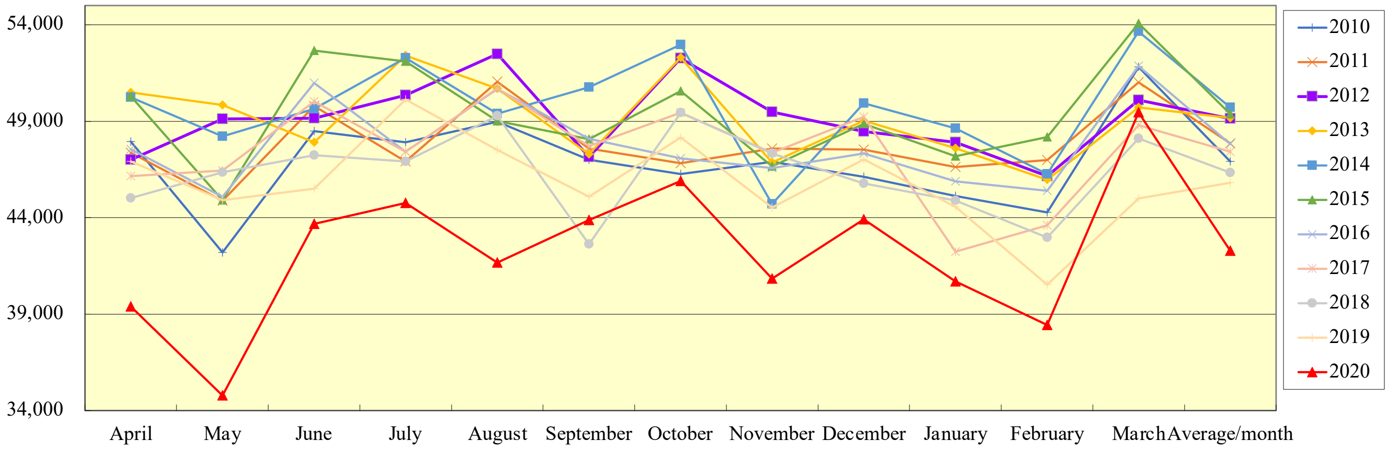

Additional Figure 15. Total outpatients per month from 2010 to 2020.

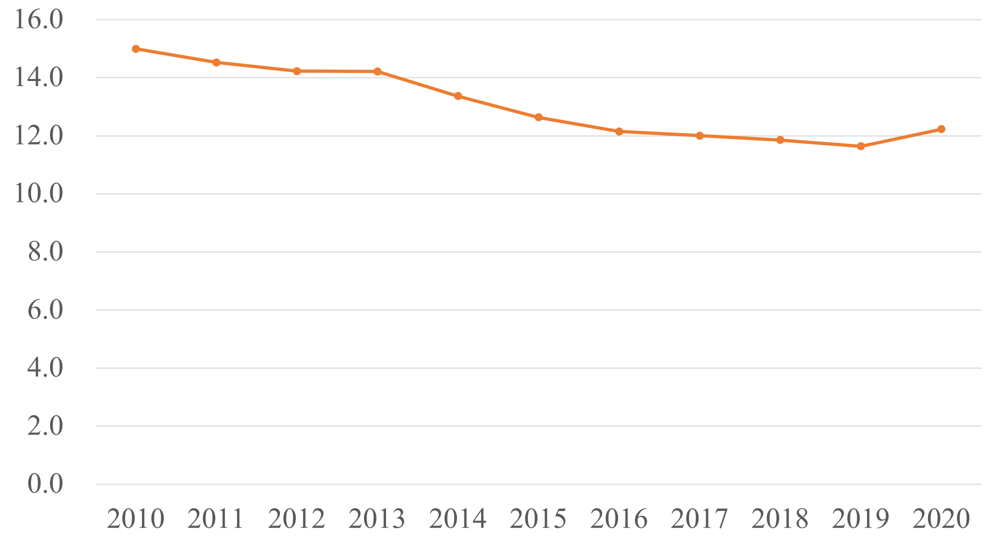

Additional Figure 16. Average hospital stay (days) from 2010 to 2020.

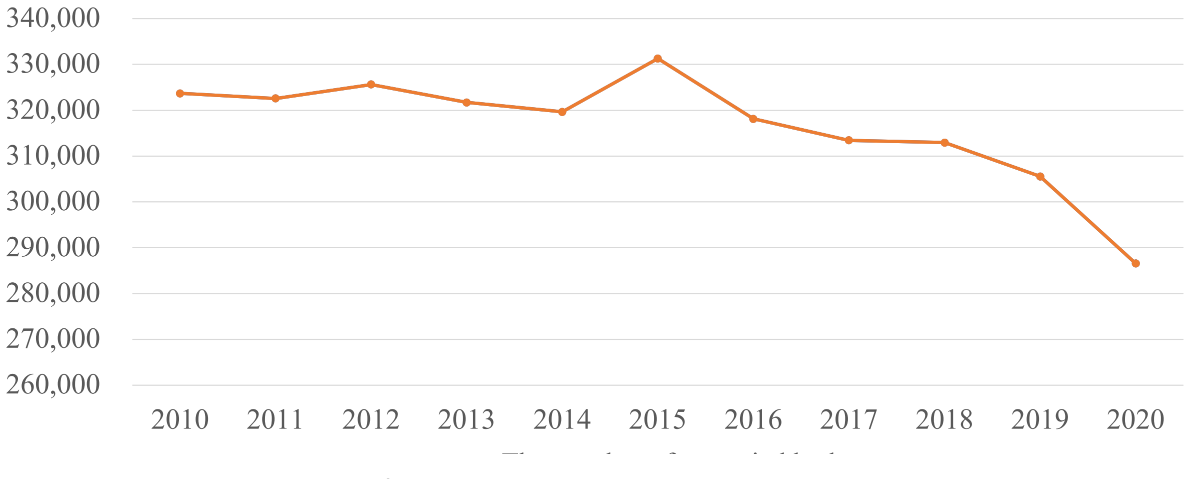

Additional Figure 17. Annual number of occupied beds from 2010 to 2020.
